# An unforgettable diagnostic journey: multimodal evaluation of jejunal diverticulitis mimicking Crohn’s disease

**DOI:** 10.1055/a-2743-2159

**Published:** 2025-12-08

**Authors:** Mohammed Abdulrasak, Mladen Makitan, Gabriele Wurm Johansson, Artur Nemeth, Anastasios Koulaouzidis, Ervin Toth

**Affiliations:** 1 174435 Department of Clinical Sciences, Lund University, Malmö, Sweden; 2 Department of Gastroenterology, Skåne University Hospital, Malmö, Sweden; 3 6174 Department of Clinical Research, University of Southern Denmark, Odense, Denmark

An 81-year-old woman with a history of rheumatoid arthritis and atrial fibrillation presented to the gastroenterology department with several months of postprandial abdominal pain, diarrhoea, and weight loss. Repeated ileocolonoscopies were normal. Magnetic resonance enterography and panenteric capsule endoscopy revealed a segment of ulcerated and narrowed small bowel ( Fig. 1 , Fig. 2 , Video 1 ), raising initial suspicion of Crohn’s disease. The terminal ileum appeared normal. Treatment with intermittent steroids resulted in no improvement. A double-balloon enteroscopy was attempted but aborted due to a cardiac event.

**Fig. 1:**
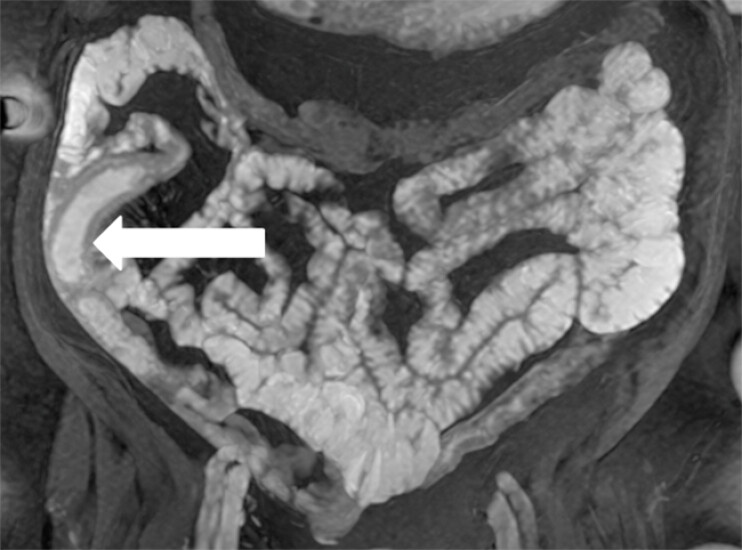
Magnetic resonance enterography showing localized small bowel wall thickening and hyperintensity, suggesting segmental inflammation in the mid-small bowel.

**Fig. 2:**
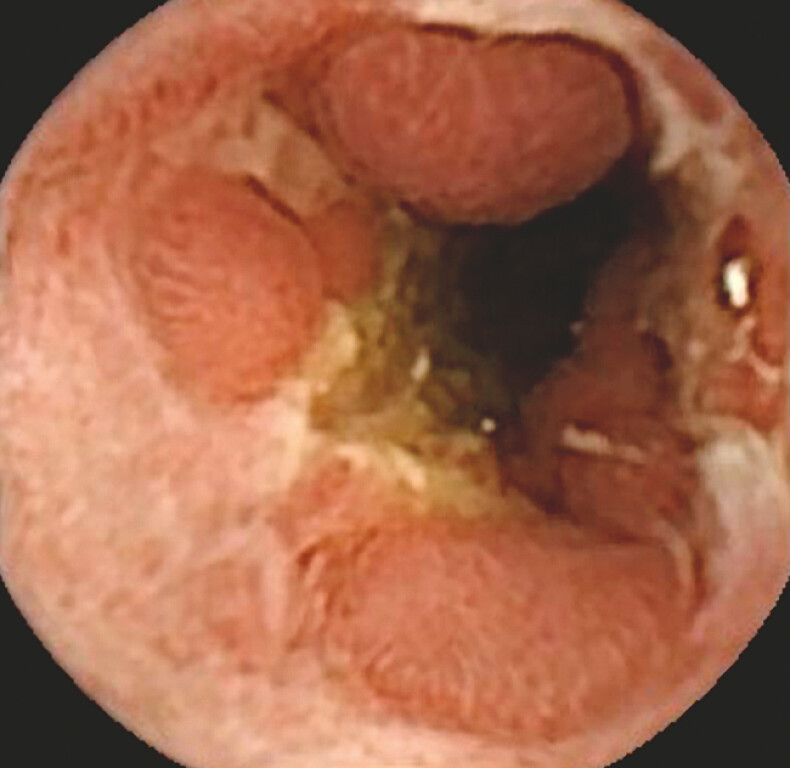
Panenteric video capsule endoscopy showing an ulcerated mid-small bowel segment with mucosal oedema and the loss of the villous pattern, findings commonly associated with Crohn’s disease.

Segment from capsule endoscopy illustrating the ulcerated region in the mid-small bowel. The capsule traverses an inflamed segment characterized by mucosal irregularity and segmental ulceration. The terminal ileum appeared endoscopically normal.Video 1

Over the following 5 years, the patient experienced intermittent symptoms and was treated empirically with steroids, without lasting improvement. Eventually, she presented with acute worsening of abdominal pain and malnutrition, prompting further investigation. Abdominal computed tomography imaging showed mural thickening and a “comb sign” in the distal jejunum/proximal ileum ( Fig. 3 ).

**Fig. 3:**
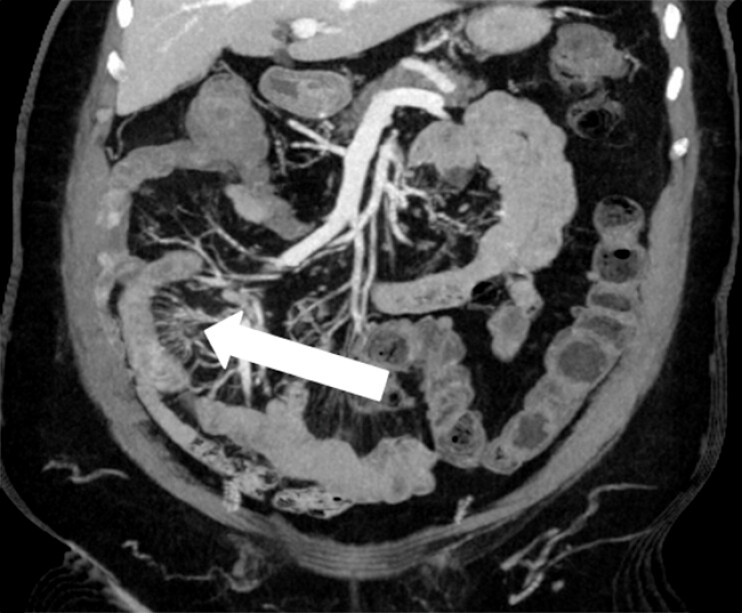
An abdominal computed tomography scan (pre-perforation) showing thickened small bowel with the “comb sign,” initially suggestive of Crohn’s disease.

Shortly after admission, signs of peritonitis emerged. A repeat abdominal computed tomography scan revealed fat stranding, suspecting local perforation alongside an 18-mm small-bowel diverticulum ( Fig. 4 ).

**Fig. 4:**
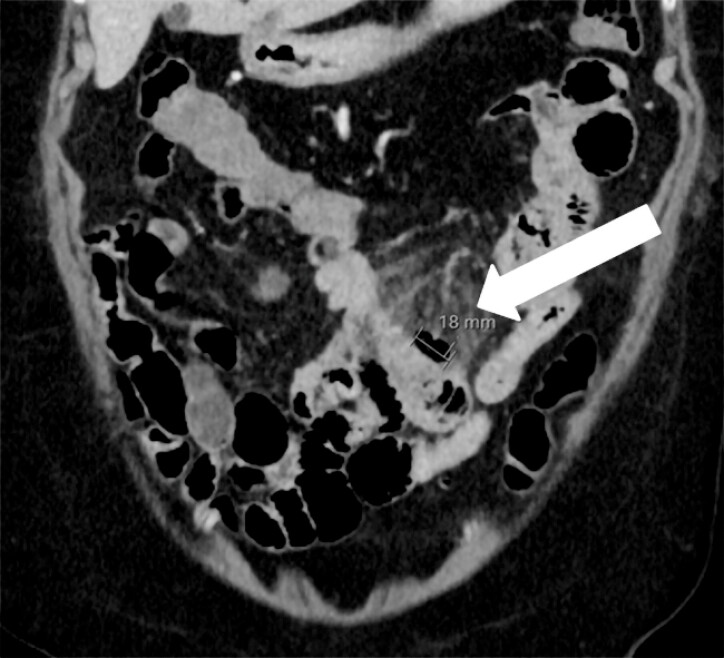
Abdominal computed tomography (post-perforation) showing fat-stranding and an 18 mm diverticulum.

Emergency laparotomy showed green pus originating from perforated jejunal diverticulitis. Subsequent macroscopic and microscopic pathological examination confirmed perforated jejunal diverticulitis ( Fig. 5 ), with no features of malignancy or inflammatory bowel disease. The patient recovered uneventfully.

**Fig. 5:**
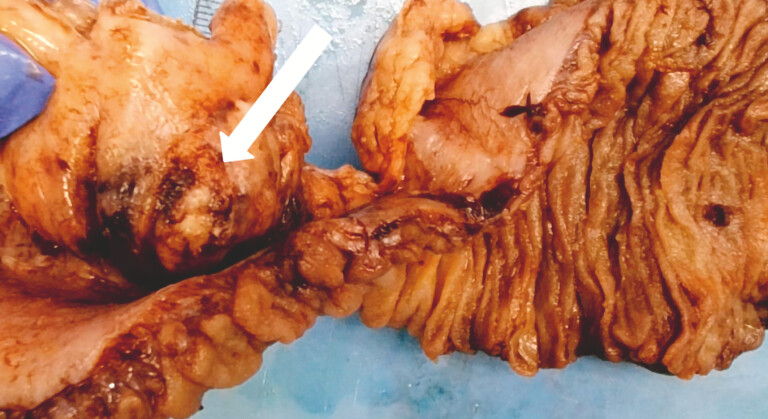
A macroscopic specimen showing an inflamed, perforated jejunal diverticulum (arrow), consistent with diverticulitis.

Jejunal diverticulitis is rare and often underrecognized but may closely mimic small bowel Crohn’s disease on capsule endoscopy and cross-sectional imaging 1 2 3 . This case highlights the importance of maintaining a broad differential and considering IBD mimics in chronic small bowel disease, particularly in older adults with atypical features or poor response to immunosuppression 4 . The VIVA mnemonic – vascular (e.g., ischemia), infectious (e.g., TB and *Yersinia* ), vasculitis (e.g., Behçet’s), autoimmune/other (e.g., NSAID enteropathy, neoplasia) – serves as a structured clinical reminder to consider IBD mimics early in the diagnostic process. The term “VIVA” also evokes the traditional oral examinations (“viva voce”) in medical training, reinforcing the intellectual rigor and diagnostic precision needed in such complex cases.

Endoscopy_UCTN_Code_CCL_1AC_2AD

## Notes

Endoscopy E-Videos https://eref.thieme.de/e-videos
*E-Videos* is an open access online section of the journal *Endoscopy* , reporting on interesting cases and new techniques in gastroenterological endoscopy. All papers include a high-quality video and are published with a Creative Commons CC-BY license. Endoscopy E-Videos qualify for HINARI discounts and waivers and eligibility is automatically checked during the submission process. We grant 100% waivers to articles whose corresponding authors are based in Group A countries and 50% waivers to those who are based in Group B countries as classified by Research4Life (see: https://www.research4life.org/access/eligibility/ ). This section has its own submission website at https://mc.manuscriptcentral.com/e-videos .
